# Hybrid immunity from bivalent vaccination and prior infection enhances humoral and innate protection against Omicron XBB.1.16 and EG.5.1.1 variants in Japan

**DOI:** 10.3389/fimmu.2026.1807238

**Published:** 2026-07-02

**Authors:** Kouki Matsuda, Shohei Yamamoto, Chihiro Motozono, Yoshiki Aritsu, Yuki Furukawa, Airi Noborio, Daisuke Takada, Hiyori Sasagawa, Yuichi Akahori, Kiyoto Tsuchiya, Hiroyuki Gatanaga, Takamasa Ueno, Norio Ohmagari, Tetsuya Mizoue, Kenji Maeda

**Affiliations:** 1 Division of Antiviral Therapy, Joint Research Center for Human Retrovirus Infection, Kagoshima University, Kagoshima, Japan; 2 Department of Epidemiology and Prevention, Center for Clinical Sciences, Japan Institute for Health Security, Tokyo, Japan; 3 Division of Infection and Immunity, Joint Research Center for Human Retrovirus Infection, Kumamoto University, Kumamoto, Japan; 4 AIDS Clinical Center, National Center for Global Health and Medicine, Japan Institute for Health Security, Tokyo, Japan; 5 Disease Control and Prevention Center, Japan Institute for Health Security, Tokyo, Japan

**Keywords:** bivalent vaccine, breakthrough infection, COVID-19, hybrid immunity, innate immunity

## Abstract

**Introduction:**

Emerging Omicron sublineages XBB.1.16 and EG.5.1.1 have caused breakthrough infections, challenging vaccine efficacy. Hybrid immunity—vaccination plus prior SARS-CoV-2 infection—augments neutralizing activity. We investigated whether innate immune responses also contribute to breakthrough prevention.

**Methods:**

We analyzed samples from a previous case-control study of 50 breakthrough infection cases and 50 controls, all adults with ≥3 mRNA vaccine doses, recruited in June 2023 during the XBB.1.5 wave in Japan. Participants were classified as hybrid immunity (prior PCR-confirmed infection or N-IgG positive), high-vaccine-induced immunity (N-IgG negative, S-IgG > 10^4^ U/mL), or low-vaccine-induced immunity (N-IgG negative, S-IgG < 10^4^ U/mL). Neutralization titers (NT_50_) were measured against ancestral SARS-CoV-2, XBB.1.16, and EG.5.1.1. Cytokines and innate markers were analyzed in serum and cell-based assays.

**Results:**

Breakthrough cases were enriched in the low-vaccine-induced immunity group, with fewer exhibiting hybrid immunity. Hybrid immunity yielded higher NT_50_ values than vaccine-only groups, but humoral immunity alone did not predict breakthrough infection. Cytokine analysis showed elevated IL-8 in hybrid non-breakthrough participants, with no differences in SARS-CoV-2-specific T-cell responses. Macrophages stimulated with the nucleocapsid protein induced IL-8, which promoted neutrophil chemotaxis. S100A8/A9, a neutrophil activation marker, correlated with IL-8 and was elevated in hybrid immunity.

**Discussion:**

Hybrid immunity may protect against Omicron XBB.1.16/EG.5.1.1 via IL-8–dependent macrophage–neutrophil interactions, highlighting a synergistic role of humoral and innate immunity.

## 1 Introduction

The emergence of immune-evasive Omicron subvariants, including XBB.1.16 (1) and EG.5.1 (2), has undermined the COVID-19 vaccines’ effectiveness. Breakthrough infections remain common despite multiple boosters, particularly during surges of highly transmissible strains (3).

Hybrid immunity, which arises from vaccination plus prior infection, has enhanced protection against multiple SARS-CoV-2 variants (4). Several studies suggest that it promotes broader, more durable antibody responses (5, 6) and may reinforce innate immune memory (7), although the underlying mechanism remains poorly understood.

By the end of 2022, Japan had introduced a bivalent mRNA vaccine targeting the spike proteins of the ancestral strain and the Omicron BA.4/5 variants. By early 2023, most of the adults over 18 years of age had received at least three doses amid the widespread exposure to Omicron XBB.1.5 (2), offering a unique opportunity to evaluate immunological protective factors before the emergence of Omicron XBB.1.16 and EG.5.1.1.

We aimed to evaluate humoral and innate immune responses in 50 breakthrough infection cases and 50 controls with ≥3 vaccine doses, sampled in June 2023 during the XBB.1.5 wave. We compared neutralizing antibody titers, cytokine profiles, and breakthrough infection rates among three groups: hybrid-immunized individuals, high vaccine responders, and low vaccine responders. These comparisons were conducted to identify cytokines correlated with hybrid immunity and to evaluate the association between specific cytokines and macrophages or neutrophils.

## 2 Materials and methods

### 2.1 Study design and ethics statement

This study included staff members of the Japan Institute for Health Security (JIHS) who participated in an ongoing COVID-19 serological survey aimed at assessing humoral immunity and infection history at the population level. The participants included both healthcare and non-healthcare workers. In our previous nested case-control study based on the eighth serological survey conducted in June 2023 (8), symptomatic COVID-19 cases (n = 206) were identified during June–September 2023 and matched to controls (1:1 propensity matching). From this previously established cohort, we randomly selected 50 matched case-control pairs (50 cases and 50 controls) and measured live-virus neutralizing activity against wild-type, XBB.1.16, and EG.5.1 variants. In the present study, we secondarily analyzed this subset to compare antibody titers and neutralization activity (**Supplementary Figure 1A**). Serum samples were stored at −80 °C, and information on COVID-19-related factors, including vaccination status, occupational infection risk, infection prevention measures, and behavioral factors, was collected via questionnaires. Prior SARS-CoV-2 infection was defined based on documented clinical history (e.g., PCR-confirmed infection) and/or seropositivity for anti-nucleocapsid (N) IgG antibodies. Vaccination status was self-reported and validated using records from the JIHS Labor Office. This study was approved by the JIHS Ethics Committee (JIHS-S-003598-11), and written informed consent was obtained from all participants. This study abided by the Declaration of Helsinki principles.

For the use of human specimens in cell-based assay, all protocols involving human participants recruited at Kumamoto University were reviewed and approved by the Institutional Review Boards of Kumamoto University (approval numbers 477). All human participants provided written informed consent.

### 2.2 Antibody testing

As part of this survey, we conducted a serological assessment to measure anti-SARS-CoV-2 nucleocapsid (N) protein antibodies and spike (S) protein antibodies were conducted. Quantitative anti-SARS-CoV-2 antibody titers were measured using the Elecsys Anti-SARS-CoV-2 S (Roche) to measure antibody levels against the receptor-binding domain of the SARS-CoV-2 spike protein. The Elecsys Anti-SARS-CoV-2 (Roche) was also used to qualitatively assess antibodies against the SARS-CoV-2 N protein (9).

### 2.3 Neutralization assay

Neutralizing activity in the serum against the wild-type (WT), Omicron XBB.1.16, and Omicron EG.5.1.1 variants was evaluated by quantifying serum-mediated inhibition of cytopathic effects (CPE) of each SARS-CoV-2 strain in HeLa_hACE2-TMPRSS2_ cells (10). The cells were obtained from the Japanese Collection of Research Bioresources Cell Bank (Osaka, Japan). Each serum sample was serially diluted five-fold in culture medium. The diluted sera were incubated with 100 50% tissue culture infectious doses (TCID_50_) of the virus at 37 °C for 30 min with a final serum dilution range of 1:40 to 1:25,000. The serum–virus mixtures were subsequently added to 96-well plates containing 1·0×10^4^ HeLa_hACE2-TMPRSS2_ cells per well. The SARS-CoV-2 strains used included a Wuhan WT strain (SARS-CoV-2^05-2N^) (11), an Omicron XBB.1.16.5 variant (SARS-CoV-2^TKYF230030/2023^, GISAID Accession ID: EPI_ISL_17775017), and an Omicron EG.5.1.1 variant (SARS-CoV-2^TKYnat14564/2023^, GISAID Accession ID: EPI_ISL_18082364). CPE levels in SARS-CoV-2–exposed cells were quantified using the Water-Soluble Tetrazolium 8 (WST-8) assay with the Cell Counting Kit-8 (Dojindo, Kumamoto, Japan, Cat# CK04) after three days of culture. The 50% neutralization titer (NT_50_) was defined as the serum dilution that achieved 50% inhibition of CPE. Each serum sample was tested in duplicate, and the mean value was used for analysis. Laboratory personnel were blinded to the samples’ status.

### 2.4 Cytokine and chemokine profiling

Human cytokines and chemokines in the serum, including interleukins (IL-1β, IL-6, IL-10, IL-12p70), tumor necrosis factor-alpha (TNF-α), interferons (IFN-α2, IFN-β, IFN-γ, IFN-λ1, IFN-λ2/3), C-X-C motif chemokine ligand 8 (CXCL8/IL-8), C-X-C motif chemokine ligand 10 (CXCL10), and granulocyte-macrophage colony-stimulating factor (GM-CSF), were analyzed using a flow cytometry-based bead immunoassay (LEGENDplex™ Human Antivirus Response panel, Biolegend, San Diego, Cat# 741270) following the manufacturer’s instructions. Quantification of S100A8/A9, a type of inflammatory mediator, was analyzed using Human Calprotectin ELISA kit (S100A8/S100A9) (Abcam, Cambridge, UK, Cat# ab267628).

### 2.5 Tetramer staining

Human peripheral blood mononuclear cells (PBMCs) were stimulated with 100 nM NF9 peptide (NYNYLYRLF, residues 448–456 of the SARS-CoV-2 spike protein, Genscript) and 20 μg of IL-8 (Peprotech, Cat# 200-08-25 μg). Cells were maintained in Roswell Park Memorial Institute (RPMI) 1640 medium (Thermo Fisher Scientific, Cat# 11875101) supplemented with 10% fetal bovine serum (FBS) and 30 U/ml recombinant human IL-2 (Peprotech, Cat# 200-02) for 14 days. The HLA-A*24:02-restricted KW9 (HIV Gag28-36: KYKLKHIVW peptide, Genscript) was used as a negative control. *In vitro*–expanded CD8^+^ T cells were stained with the QuickSwitch™ Quant HLA-A*24:02 Tetramer Kit–Phycoerythrin (PE; MBL International Corporation, Cat# TB-7302-K1) following the manufacturer’s protocol. The cells were first washed and stained with tetramers for 30 min. The surface was subsequently stained with the following antibodies: CD3 AF532 (clone: UCHT1) (eBioscience, Cat# 58-0038-42), CD8 Pacific Blue (clone: HIT8a) (BioLegend, Cat# 300928), and CD4 BV750 (clone: SK3) (BioLegend, Cat# 344644). Dead cells were stained with 7-aminoactinomycin D. Following incubation for 20 min, the cells were fixed with 1% paraformaldehyde (Nacalai Tesque, Cat# 09154-85), and the levels of tetramer^+^ CD8^+^ T cells were analyzed by flow cytometry using a Cytek Northern Lights flow cytometer (Cytek Japan), followed by analysis using FlowJo v10 software (BD Biosciences).

### 2.6 Differentiation of macrophages

Human monocyte-derived macrophages (MDMs) were established as reported previously (12) with minor modifications. PBMCs were isolated from healthy donors by Ficoll-Paque density gradient centrifugation. Cells were seeded in a 12-well plate containing RPMI 1640 (Thermo Fisher Scientific) supplemented with 10% FBS, 50 U/mL penicillin, and 100 µg/mL streptomycin and cultured at 37°C in 5% CO_2_. Monocytes were differentiated into macrophages over 5 days in the presence of 100 ng/mL GM-CSF (Biolegend, Cat# 576302). Differentiation was assessed by flow cytometry analysis of CD11b, CD14 and CD68 expression as described previously (13). In brief, GM-CSF unstimulated and stimulated cells were stained with APC anti-human CD11b mAb (clone: ICRF44) (Biolegend, Cat# 301310) and/or FITC anti-human CD14 mAb (clone: HCD14) (Biolegend, Cat# 325603) for 30 min on ice. Then, cells were fixed with 1% paraformaldehyde/PBS for 15 min and permeabilized with Flow Cytometry Perm Buffer (TONBO Biosciences, San Diego, CA). After 5 min of incubation at room temperature, the cells were stained with FITC anti-human CD68 mAb (clone: Y1/82A) (Biolegend, Cat# 333805) for 30 min on ice. Cells were subsequently analyzed using a CytoFLEX flow cytometer (Beckman Coulter, Brea, CA). The acquired data were analyzed with FlowJo software. The resulting cells were treated with 1 µg/mL SARS-CoV-2 spike S1 protein (RayBiotech, Inc., Norcross, GA, Cat# 230-30162) and SARS-CoV-2 nucleocapsid protein (RayBiotech, Inc., Cat# 230-30164) for 24 hours.

### 2.7 Gene expression analysis

Total RNA was isolated using an RNeasy mini kit (Qiagen, Helden, Germany, Cat# 74104), following the manufacturer’s instructions. Complementary DNA (cDNA) was synthesized using PrimeScript RT Master Mix (Takara-bio, Shiga, Japan, Cat# RR036A). Quantitative real-time PCR analysis for IL-6 and CXCL8/IL-8 mRNA was conducted with PowerUp™ SYBR Green Master Mix (Applied Biosystems, Foster City, CA, Cat# A25742). The oligonucleotide primers used were as follows: 5’-AGACAGCCACTCACCTCTTCAG-3’ (forward) and 5’-TTCTGCCAGTGCCTCTTTGCTG-3’ (reverse) for *IL-6* (14), 5’-TCTGCAGCTCTGTGTGAAGGT-3’ (forward), and 5’-TGAATTCTCAGCCCTCTTCAA-3’ (reverse) for *CXCL8/IL-8* (15), 5’-GAACTGTACGCTGTACCTGCA-3’ (forward) and 5’-TTGATGGCCTTCGATTCTGGA-3’ (reverse) for *CXCL10* (16), and 5′-GCGAGAAGATGACCCAGATC-3′ (forward) and 5′-CCAGTGGTACGGCCAGAGG-3’ (reverse) for *β-actin* (17). Fold variations in gene expression levels were calculated using the ΔΔC_T_ method. Triplicate measurements were obtained for each sample.

### 2.8 Differentiation of neutrophil-like cells

A neutrophil-like cell model was established as described previously (18, 19) with minor modifications. HL-60 cells, a promyelocytic leukemia cell line, were cultured in RPMI 1640 supplemented with 10% FBS and penicillin/streptomycin at 37°C in 5% CO_2_. HL-60 cells (5×10^5^ cells/ml) were treated with 1·3% dimethyl sulfoxide (DMSO) for 4 days to induce their differentiation into a neutrophil-like phenotype (dHL-60 cells). Differentiation was assessed by flow cytometry analysis of CD11b surface expression as described previously (13). In brief, HL-60 and dHL-60 cells were stained with APC anti-human CD11b mAb (clone: ICRF44) (Biolegend, Cat# 301310) for 30 min on ice. Cells were subsequently analyzed using a CytoFLEX flow cytometer (Beckman Coulter, Brea, CA). The acquired data were analyzed with FlowJo software.

### 2.9 Chemotaxis assay

The chemotactic transwell assay of dHL-60 neutrophil-like cells was analyzed in the presence of recombinant human IL-8 (Biolegend, Cat# 574204). A total of 500 µL of dHL-60 cells (2×10^6^ cells/mL) were loaded into the membrane of the upper chamber of a 12-well Transwell plate (Transwell^®^-12 Well Plate, polyester membrane, pore size = 3·0 µm, Corning Inc., Corning, NY, USA, Cat# 3462). The lower chamber of each Transwell contained 1,500 µL of cell culture medium containing 0–100 ng/mL of rhIL-8. Transwell plates were incubated in a 5% CO_2_ incubator at 37°C for 3 hours. Following incubation, cells that migrated into the lower chambers were collected and counted using trypan blue staining. Relative chemotaxis was calculated by normalizing the number of migrated cells in each condition to that of the untreated control, which was set to 1.0.

### 2.10 Statistical analysis

Analysis of differences between groups were performed using the Mann–Whitney U test, the unpaired t-test, or one-way ANOVA. Data are presented as medians with interquartile ranges. Anti-N IgG titers, IL-8 production levels, and S100A8/A9 production levels were log_10_-transformed before the correlation analysis to approximate a normal distribution and were assessed using Pearson’s r test. Statistical significance was set at P < 0·05. GraphPad Prism version 8 (GraphPad Software, La Jolla, CA) was used for all analyses.

## 3 Results

### 3.1 Hybrid immunity and bivalent vaccination sustain neutralization against Omicron variants

**Figure 1:**
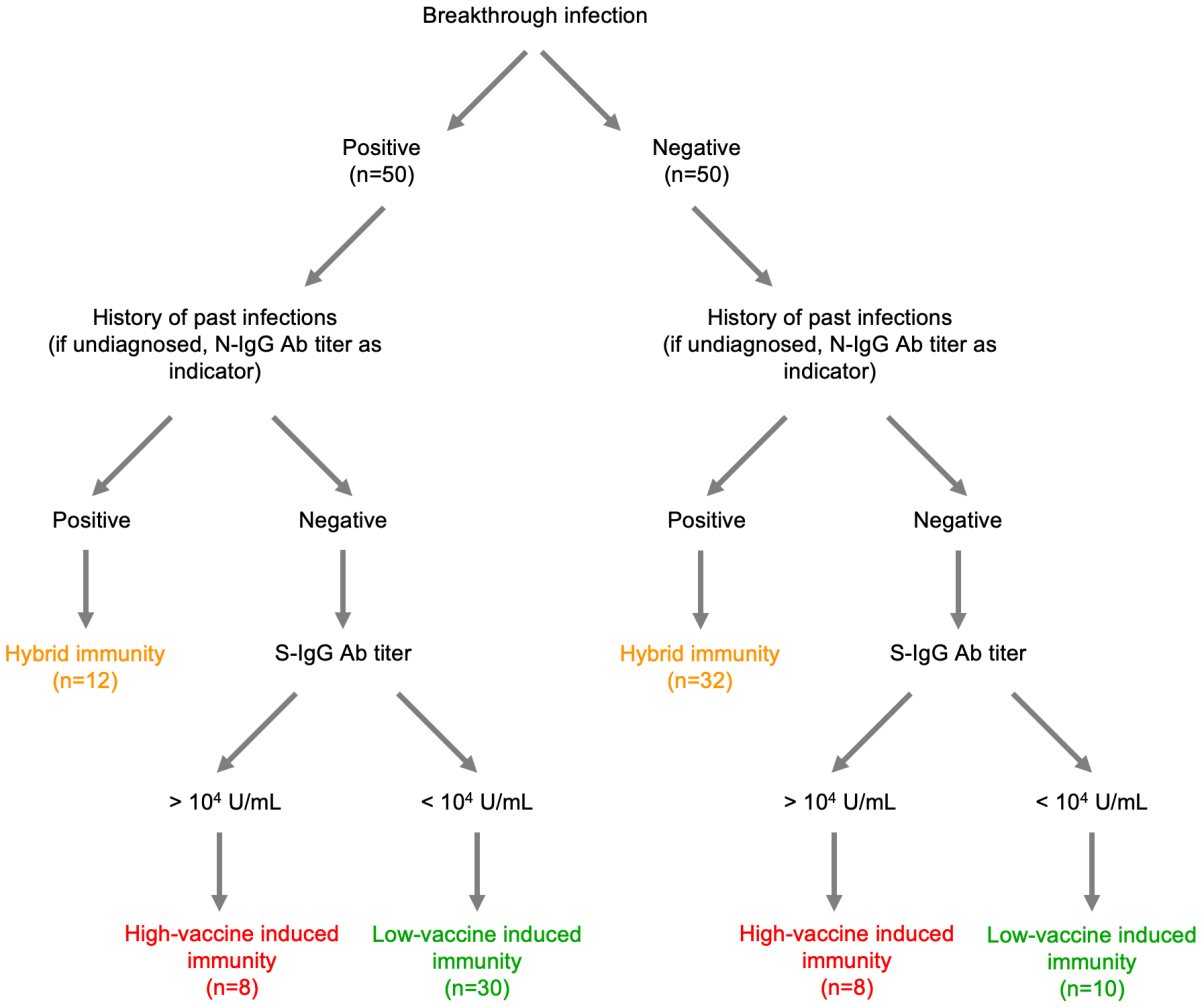
Breakthrough infection status and immunological classification of participants. Schematic of the participants’ classification. Participants were classified into six groups: breakthrough hybrid immunity (n = 12), with prior PCR-confirmed infection or N-IgG positivity; breakthrough high-vaccine-induced immunity (n = 8), N-IgG negative with S-IgG > 10^4^ U/mL; breakthrough low-vaccine-induced immunity (n = 30), N-IgG negative with S-IgG < 10^4^ U/mL; non-breakthrough hybrid immunity (n = 32); non-breakthrough high-vaccine-induced immunity (n = 8); and non-breakthrough low-vaccine-induced immunity (n = 10).

Participants were classified according to prior SARS-CoV-2 infection status and vaccine-induced antibody responses, as illustrated in **Figure 1**. First, individuals were categorized based on prior infection, defined by anti-N IgG positivity and/or documented clinical diagnosis. Participants with prior infection were assigned to the hybrid immunity group. Among participants without evidence of prior infection (N-IgG negative), individuals were further stratified based on anti-S IgG titers into high (S-IgG > 10^4^ U/mL) and low (S-IgG < 10^4^ U/mL) vaccine-induced immunity groups, as shown in **Supplementary Figure 1B**. Within each group, participants were subsequently classified according to the presence or absence of breakthrough infection, resulting in six subgroups. The number of individuals with and without breakthrough infection in each group was as follows: hybrid immunity (12 vs 32), high-vaccine-induced immunity (8 vs 8), and low-vaccine-induced immunity (30 vs 10). Among breakthrough infection cases, the proportion of the low vaccine-induced immunity group was highest (60%), whereas among non-breakthrough infection cases, the proportion of the hybrid immunity group was highest (64%). A chi-square test demonstrated a significant difference in the distribution of breakthrough infection status across the three groups (P < 0·0001) (**Table 1**). In our previous study, we only compared neutralizing antibody responses according to the presence or absence of breakthrough infections (8). In this study, we additionally incorporated hybrid immunity and vaccine-induced immunity into the comparison. First, we compared neutralizing activity among the three groups in the entire cohort using hybrid immunity, high-vaccine-induced immunity, and low-vaccine-induced immunity. The hybrid immunity group exhibited significantly higher NT_50_ values than the values of low-vaccine-induced immunity group against WT, Omicron XBB.1.16, and EG.5.1.1 (P < 0·001) (**Supplementary Figure 2**). Additionally, the hybrid immunity group exhibited significantly higher NT_50_ values against Omicron XBB.1.16 than those of the high-vaccine-induced immunity group (P < 0·05) (**Supplementary Figure 2**). These results highlight the broad efficacy of the hybrid response against antigenically distinct Omicron variants.

**Table 1:** Proportion of breakthrough infections across immune status group.

Immune status	Breakthrough (+) *^a^*	Breakthrough (-) *^a^*	P value *^b^*
Hybrid immunity	12 (24%)	32 (64%)	
High-vaccine induced immunity	8 (16%)	8 (16%)	
Low-vaccine induced immunity	30 (60%)	10 (20%)	< 0.0001

Breakthrough infection was defined as SARS-CoV-2 infection occurring after vaccination. The proportions of individuals with breakthrough infection were compared among the hybrid immunity group, high vaccine-induced immunity group, and low vaccine-induced immunity group. *^a^* Data are presented as number (percentage) of individuals in each group. *^b^* Statistical significance was assessed using the chi-square test.

To determine whether the vaccine response is associated with the maintenance of neutralizing activity, we compared the number of vaccine doses with the occurrence of breakthrough infections. Comparing the proportion of participants who received five or more doses between breakthrough infection (+) and breakthrough infection (-) groups showed 8·3% and 25·0% in the hybrid immunity group, 62·5% and 87·5% in the high-vaccine-induced immunity group, and 23·3% and 20·0% in the low-vaccine-induced immunity group, respectively. A trend towards higher vaccination doses was observed in groups that experienced no breakthrough infection (**Supplementary Figure 3A**). However, subsequent analysis of breakthrough infections and humoral immunity revealed no significant differences between the group that experienced breakthrough infections and the group that did not, particularly regarding S-IgG levels (**Supplementary Figure 3B**) and neutralizing antibody titers (**Supplementary Figure 3C**). These findings suggest that humoral immunity alone does not account for the observed protective effect.

**Figure 2:**
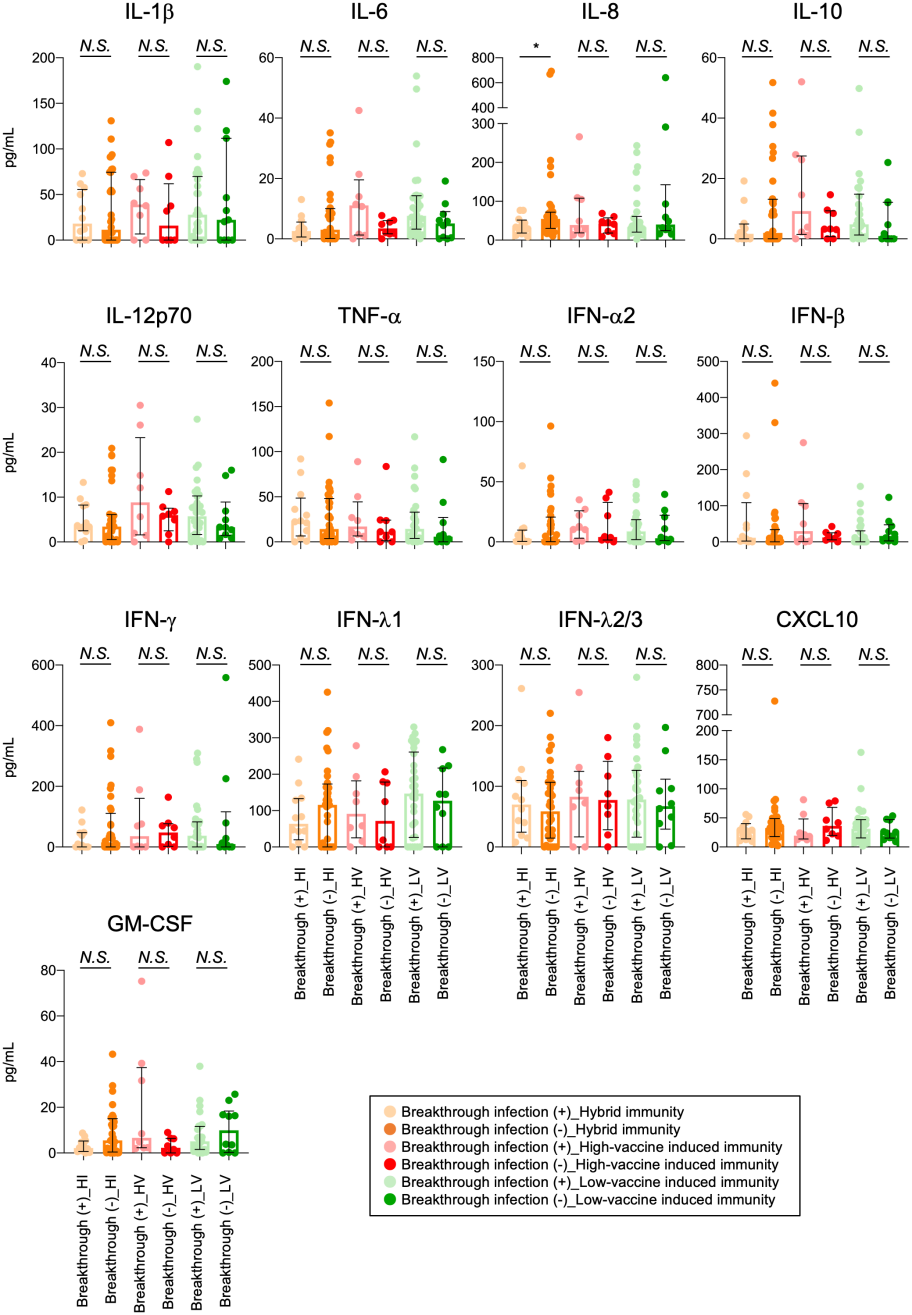
Profiling of serum cytokines and chemokines. Serum concentrations of cytokines and chemokines were compared across groups defined by immune status (hybrid immunity [HI], high-vaccine-induced immunity [HV], and low-vaccine-induced immunity [LV] and breakthrough infection status (with or without breakthrough infection), resulting in six subgroups. Bar graphs indicate the median titers, and the I-shaped bars denote interquartile ranges. Statistical comparisons between breakthrough infection-positive and -negative individuals within each immune group were performed using the Mann–Whitney U test (N.S.: not significant; *P < 0·05).

Multiplex cytokine analysis revealed that no statistically significant differences were observed across all six groups when assessed by one-way ANOVA or the Kruskal–Wallis test (results not shown). Given the heterogeneity in immune background, we performed stratified analyses according to immune status. Within each group, comparisons between individuals with and without breakthrough infection were conducted. Serum IL-8 levels were significantly elevated in the hybrid immunity group, particularly in individuals who did not experience breakthrough infection (P < 0·05) (**Figure 2**), whereas no significant differences were detected in high or low-vaccine-induced immunity groups. These findings suggests that enhanced innate immune activation is associated with reduced breakthrough infection. However, as T cell responses were not directly evaluated in this cohort, the contribution of adaptive immunity cannot be excluded.

### 3.2 Nucleocapsid proteins promote inflammatory responses in macrophages

**Figure 3:**
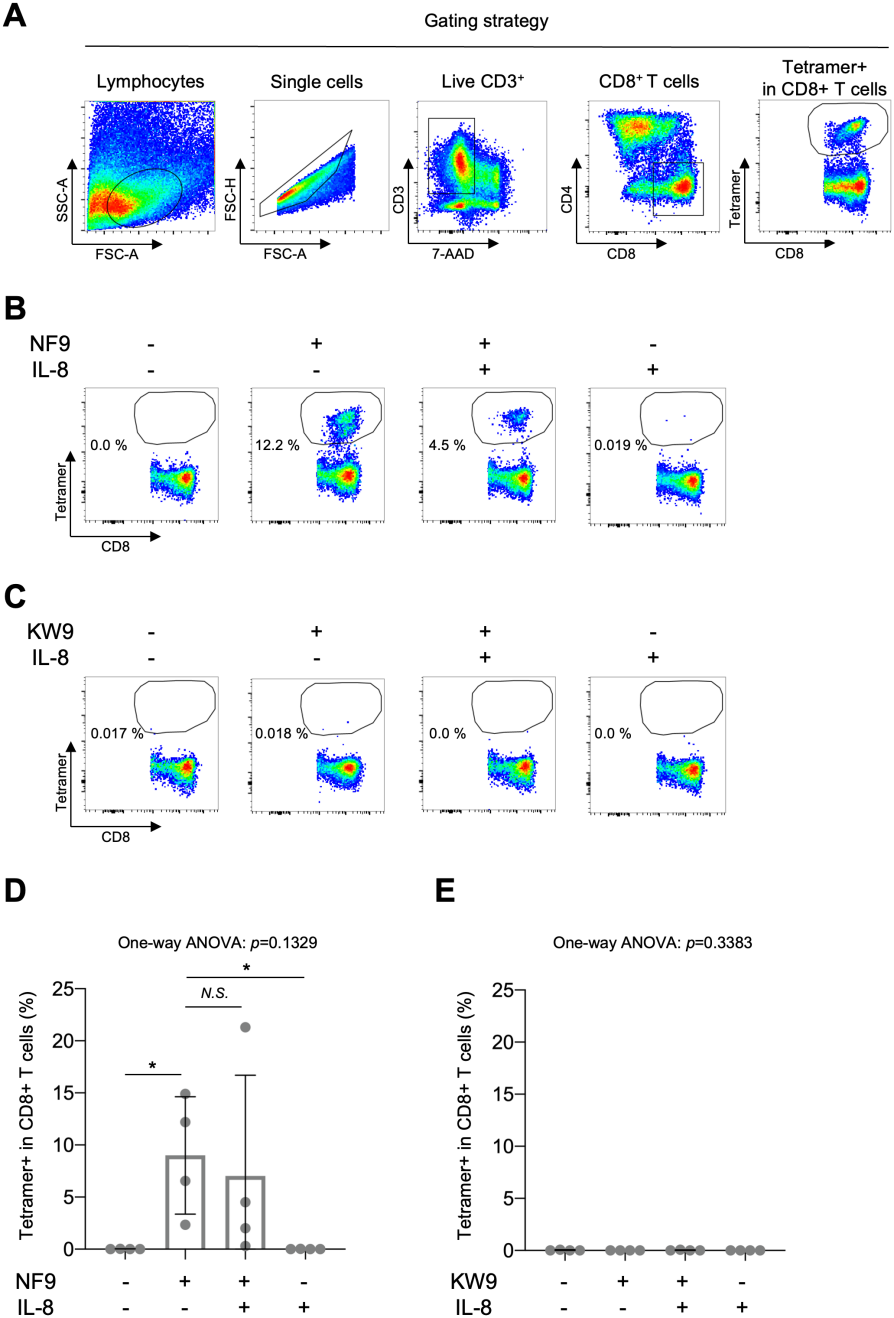
The effect of IL-8 on the induction of SARS-CoV-2-specific CD8^+^ T cells. **(A)** Representative flow cytometric gating strategy for the analysis of tetramer-positive CD8-positive T cells. **(B)** Representative flow cytometric analysis of tetramer-positive CD8-positive T cell induction following stimulation with the NF9 peptide (HLA-A*24:02-restricted SARS-CoV-2 spike epitope) in the presence or absence of IL-8 treatment. **(C)** Representative flow cytometric analysis of tetramer-positive CD8-positive T cell induction following stimulation with the KW9 peptide (negative control peptide) in the presence or absence of IL-8 treatment. **(D)** Summary of NF9 peptide-induced tetramer-positive CD8-positive T cell frequencies shown in **(B, E)** Summary of KW9 peptide-induced tetramer-positive CD8-positive T cell frequencies shown in **(C)**. Data were obtained from four independent donors. Statistical significance was determined using the one-way ANOVA and the unpaired t-test for pairwise comparisons (N.S.: not significant; *P < 0·05).

To validate the association between IL-8 production and cellular immunity, we first considered its potential impact on adaptive responses. Although IL-8 is primarily recognized as a neutrophil chemoattractant, accumulating evidence suggests that it can also influence T-cell activation and recruitment under inflammatory conditions. Given that hybrid immunity is associated with enhanced SARS-CoV-2-specific CD8 T cell responses in prior studies (20), we hypothesized that elevated IL-8 might modulate CD8 T cell induction. To investigate this, PBMCs isolated from HLA-A*24:02-positive vaccinated individuals were stimulated using the NF9 peptide (NYNYLYRLF, residues 448–456 of the SARS-CoV-2 spike protein), a representative HLA-A24*24:02-restricted SARS-CoV-2 spike epitope. The KW9 peptide (KYKLKHIVW, residues 28–36 of the HIV gag protein), presented by the same HLA haplotype, was used as a negative control peptide (21). SARS-CoV-2-specific CD8 T cell induction was evaluated by tetramer staining and flow cytometric analysis, and representative gating strategies are shown in **Figure 3A**. The percentages of tetramer^+^ CD8^+^ T cells were quantified and compared between conditions with or without IL-8 treatment (**Figures 3B–E**). However, no significant differences in tetramer^+^ CD8^+^ T cell induction were observed following IL-8 treatment.

**Figure 4:**
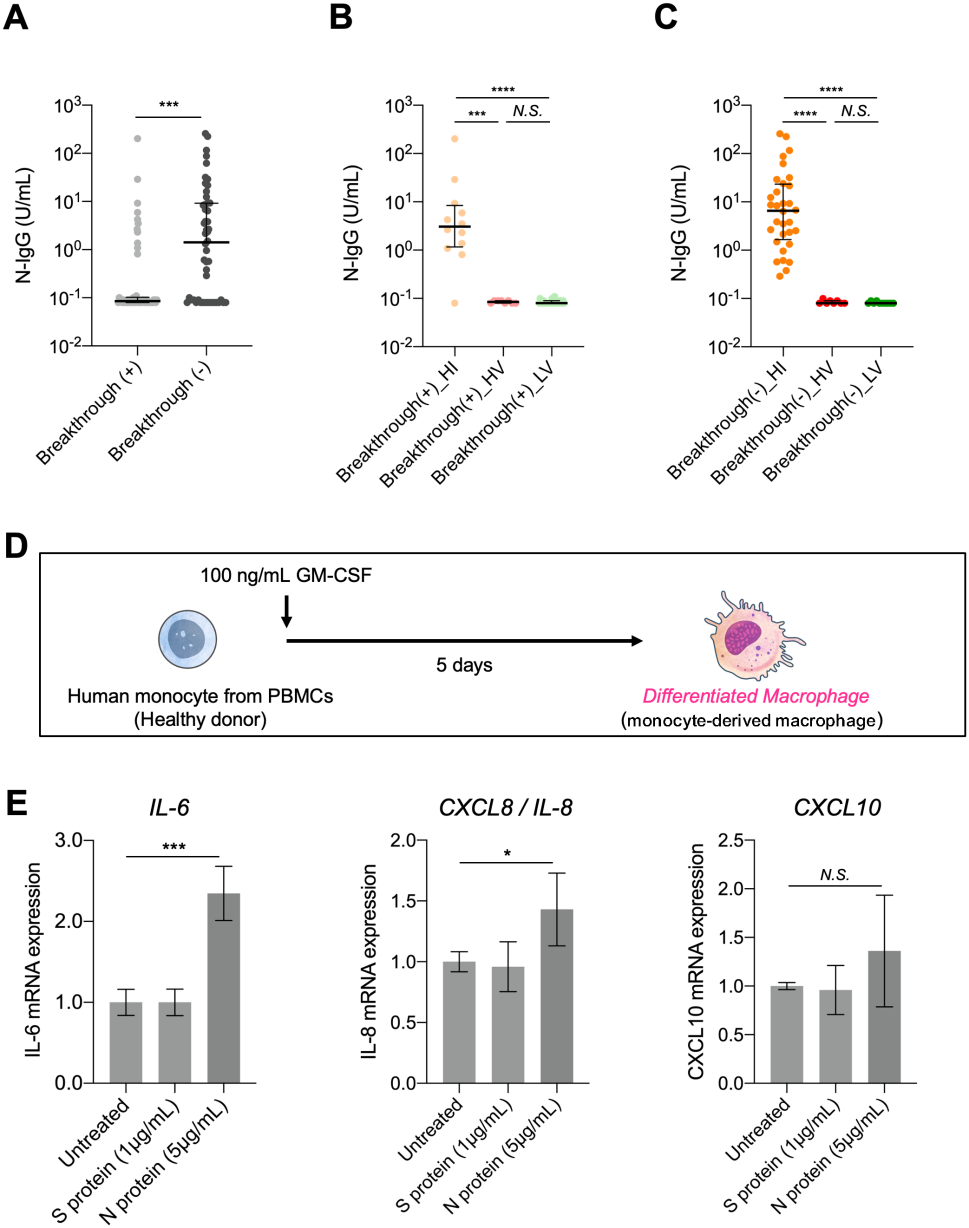
Nucleocapsid proteins promote inflammatory responses in macrophages. **(A)** Comparison of anti-nucleocapsid antibody titers among 50 breakthrough infection cases and 50 non-breakthrough infection cases. **(B)** Comparison of anti-nucleocapsid antibody titers among the three groups with breakthrough infections. **(C)** Comparison of anti-nucleocapsid antibody titers among the three groups with no breakthrough infections. Statistical significance was determined using the Mann–Whitney U test (N.S.: not significant; ***P < 0·001; ****P < 0·0001). **(D)** The experimental scheme of macrophage differentiation. Human monocytes from healthy donors were differentiated following 5 days of treatment with 100 ng/mL GM-CSF. **(E)** Gene expression of IL-6, CXCL8/IL-8, and CXCL10 mRNA of differentiated macrophages treated with recombinant SARS-CoV-2 viral proteins for 24 hours. Bar graphs indicate the means, and the I-shaped bars denote standard deviations. Statistical significance was determined using the unpaired t-test (N.S.: not significant; *P < 0·05; ***P < 0·001). HI, Hybrid Immunity; HV, High-vaccine-induced immunity; LV, Low-vaccine-induced immunity. Aspects of figure include illustrations from NIH BioArt Source (bioart.niaid.nih.gov/bioart/358, bioart.niaid.nih.gov/bioart/313).

Macrophages have been implicated in SARS-CoV-2 disease severity. Prior studies have shown that nucleocapsid antigen and anti-N IgG promote IL-6 production in monocyte-derived macrophages, contributing to the cytokine storm observed in COVID-19 (12, 22). Anti-N IgG titers were significantly higher in participants who did not experience breakthrough infections than in those who experienced breakthrough infections (**Figure 4A**). The hybrid immunity group exhibited significantly higher anti-N IgG titers than those of the other groups (**Figures 4B, C**).

To clarify the mechanisms underlying the increase in IL-6 and IL-8, we investigated whether viral components directly induce their production in MDMs generated from PBMCs of healthy donors (**Figure 4D**). MDMs were induced with GM-CSF, and the expression of CD11b, CD14, CD68 was assessed by flow cytometry after 5 days (**Supplementary Figure 4A**). We observed a significant increase in CD11b and CD68 expression and a decrease in CD14 expression; since the majority of cells constituted the CD11b^+^CD14^-^ and CD11b^+^CD68^+^ cell subsets, we confirmed that the MDMs had been successfully differentiated (**Supplementary Figures 4B–F**). *In vitro* stimulation of MDMs with recombinant SARS-CoV-2 nucleocapsid protein significantly upregulated IL-6 and IL-8 mRNA expression; this aligns with prior reports (**Figure 4E**). However, CXCL10, another chemokine implicated in cell migration and COVID-19 severity, exhibited an increasing trend; this change was not statistically significant (**Figure 4E**). These findings suggest that prior infection may have triggered sustained activation of macrophages, contributing to an inflammatory environment.

### 3.3 Neutrophils activated by IL-8 may have contributed to the prevention of breakthrough infection

Activated macrophages secrete many chemokines that promote inflammatory responses and immune responses (23). These chemokines recruit other immune cells, such as neutrophils, monocytes, and lymphocytes, to the site of inflammation, where they facilitate pathogen clearance and tissue repair (24). Within the innate immune system, neutrophils are particularly responsive to chemokine signals, as they gather at the site of inflammation to phagocytose and kill pathogens (25). By engulfing viral particles and apoptotic bodies containing viruses, neutrophils help remove viruses and prevent viral replication and infection in surrounding cells (26). During SARS-CoV-2 infection, excessive infiltration of neutrophils and macrophages in the lungs has been implicated in fatal cytokine storms (27). Conversely, moderate neutrophil activation exerts antiviral activity against SARS-CoV-2 through NETosis, contributing to the clearance of infected cells (28).

**Figure 5:**
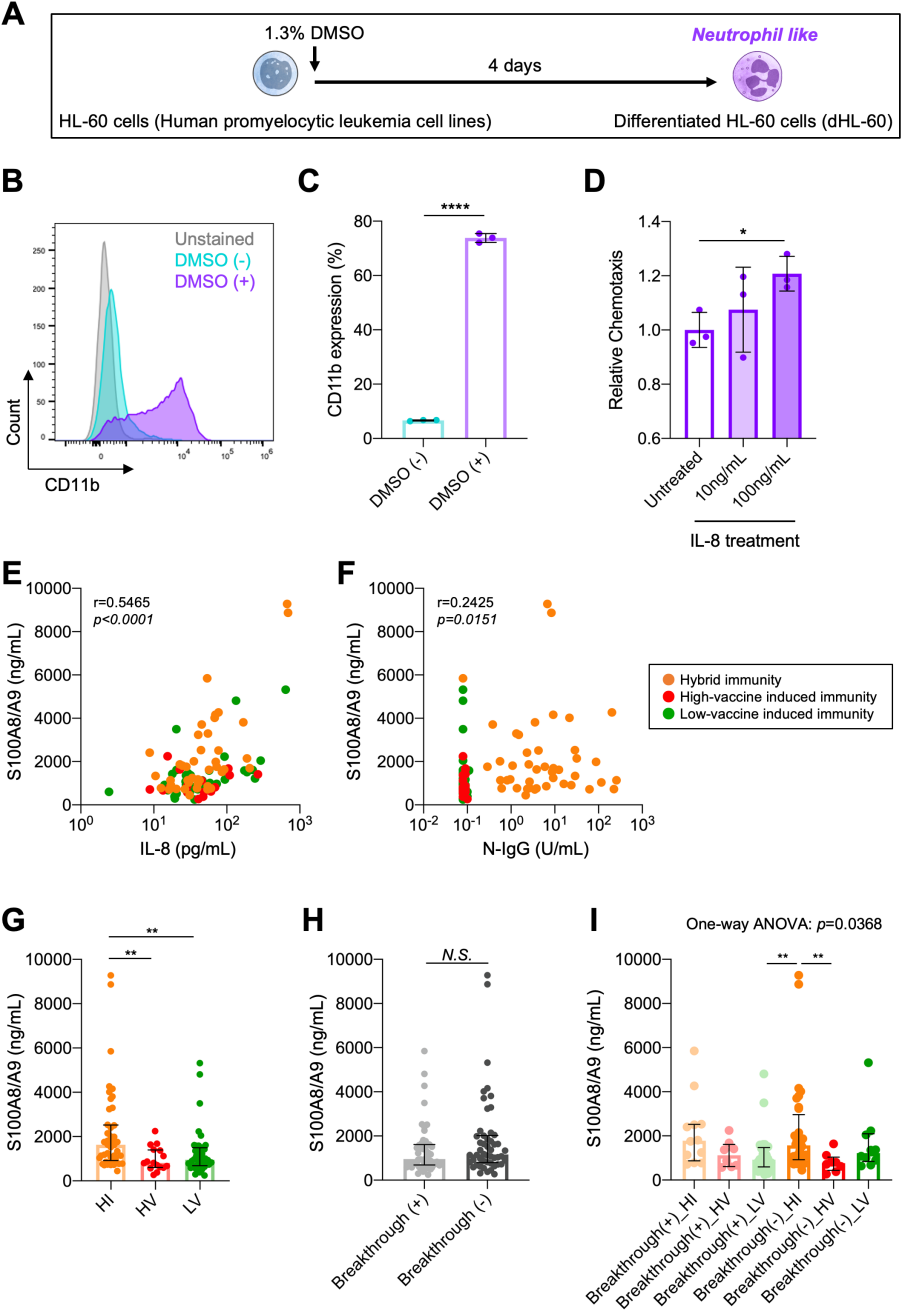
IL-8-mediated neutrophil activation may have contributed to the prevention of breakthrough infection. **(A)** The experimental schematic of neutrophil differentiation. Human promyelocytic leukemia cell line, HL-60 cells, were differentiated following 4 days of treatment with 1.3% DMSO. **(B)** Representative histogram of CD11b expression in unstained, unstimulated HL-60 and stimulated cells (dHL-60). **(C)** Comparison of the proportion of CD11b^+^ cells between HL-60 and dHL-60 cells. **(D)** Chemotaxis assay of dHL-60 cells treated with IL-8. Relative chemotaxis was calculated by setting the number of migrated cells in the untreated control condition to 1.0. Bar graphs indicate the mean, and the I-shaped bars denote standard deviations. Statistical significance was determined using the unpaired t-test (*P < 0·05; ****P < 0·0001). **(E)** Correlation between S100A8/A9 and IL-8 levels in serum. **(F)** Correlation between S100A8/A9 levels and anti-nucleocapsid antibody titers in serum. Statistical significance was determined using Pearson’s (r) **(G)** Comparison of S100A8/A9 serum levels among the three groups. **(H)** Comparison of S100A8/A9 serum levels among 50 breakthrough infection cases and 50 non-breakthrough infection cases. **(I)** Comparison of S100A8/A9 serum levels among the six groups. Bar graphs indicate the median titers, and the I-shaped bars denote interquartile ranges. Statistical significance was determined using the one-way ANOVA and the Mann–Whitney U test for pairwise comparisons (N.S.: not significant; *P < 0·05; **P < 0·01). HI, Hybrid Immunity; HV, High-vaccine-induced immunity; LV, Low-vaccine-induced immunity. Aspects of figure include illustrations from NIH BioArt Source (bioart.niaid.nih.gov/bioart/84, bioart.niaid.nih.gov/bioart/379).

Given the role of IL-8 as a potent chemokine, we investigated neutrophil functions in the hybrid immunity context. *In vitro* experiments confirmed the differentiation of HL-60 cells (human promyelocytic leukemia cell line) into neutrophil-like cells (dHL-60) and the expression of CD11b (**Figures 5A–C**). IL-8 stimulation was demonstrated to enhance neutrophil chemotaxis (**Figure 5D**). Moreover, plasma levels of the neutrophil activation marker S100A8/A9 were positively correlated with IL-8 concentrations (r = 0·5465, P < 0·0001) (**Figure 5E**). In contrast, the association with anti-N IgG titers was weak, although statistically significant (r = 0·2425, P = 0·0151) (**Figure 5F**). Notably, S100A8/A9 plasma levels were significantly higher in the hybrid immunity group than in the other groups (P < 0·01) (**Figure 5G**). Participants who did not experience breakthrough infections tended to have higher S100A8/A9 blood levels than those who did; however, the difference was not statistically significant (**Figure 5H**). In the analysis of the six groups, which considered breakthrough infections, a one-way ANOVA revealed significant differences (P = 0·0368). To identify the cause of these differences, we focused on biologically relevant comparisons and performed predefined pairwise comparisons. Notably, within the hybrid immunity group, individuals who did not experience breakthrough infections exhibited higher blood concentrations of S100A8/A9 than those of individuals in the low-vaccine-induced immunity group who experienced breakthrough infections (**Figure 5I**). Overall, these results suggest that enhanced IL-8 production and neutrophil activation may constitute an innate immune axis that contributes to protection against Omicron variant breakthrough infections in hybrid-immunized individuals.

The distribution of baseline characteristics, including vaccine dose, was comparable between cases and controls (**Supplementary Table 1**). To assess the potential impact of vaccine dose on these findings, we examined correlations between vaccine dose and immunological parameters (**Supplementary Table 2**). No significant correlations were observed between vaccine dose and IL-8 levels or neutrophil activation markers, whereas a weak but statistically significant negative correlation was observed with N-IgG titers (r = −0·2057, P = 0·04).

## 4 Discussion

Our study demonstrates that hybrid immunity from bivalent vaccination and prior SARS-CoV-2 infection confers superior protection against Omicron variants, including XBB.1.16 and EG.5.1.1. The hybrid immunity group exhibited the highest neutralizing antibody titers against these variants and demonstrated characteristics specific to the activation of innate immunity, indicating a dual protective mechanism.

Although vaccine-induced S-IgG levels were robust among the high-vaccine-induced immunity group, neutralization titers against Omicron variants were significantly lower than those in the hybrid immunity group. These results align with previous findings indicating that natural infection imprints a broader antibody profile, potentially owing to epitope exposure beyond the spike protein, including exposure to the nucleocapsid protein (29). Moreover, our data imply that S-IgG titers alone may be insufficient as indicators of protection against immune-evasive variants.

**Figure 6:**
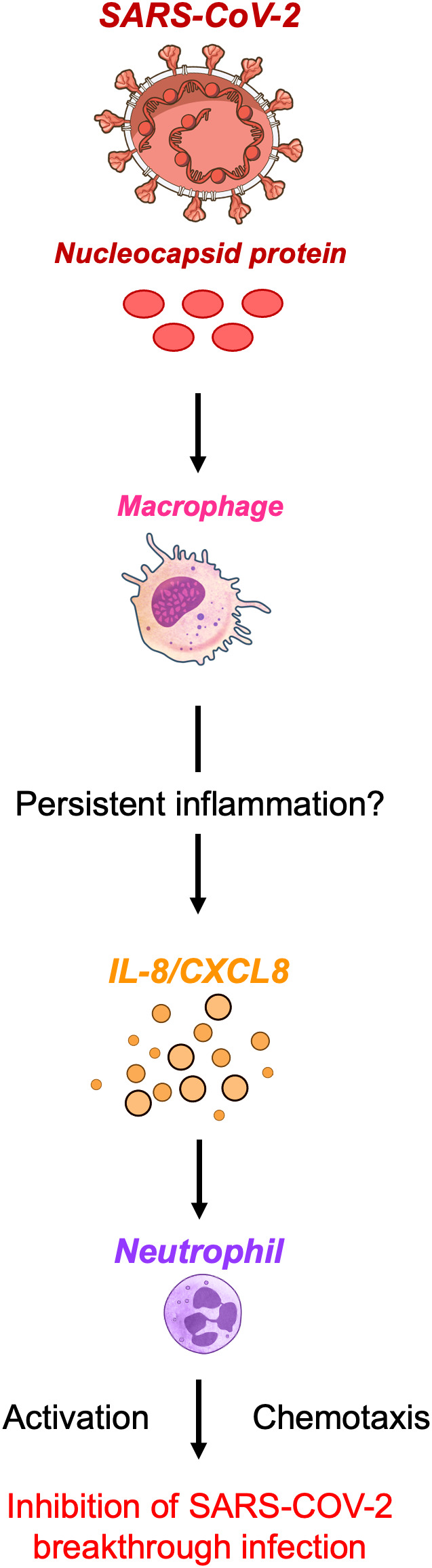
The mechanisms of IL-8-mediated immune activation of neutrophils to protect against SARS-CoV-2 breakthrough infection. Prior SARS-CoV-2 infection may induce sustained innate immune activation characterized by elevated IL-8 production and neutrophil activation. *In vitro* experiments demonstrated that SARS-CoV-2 nucleocapsid proteins stimulated IL-8 production in monocyte-derived macrophages (MDMs), which subsequently enhanced neutrophil chemotaxis. These findings suggest that the IL-8-neutrophil axis may associated with protection against SARS-CoV-2 breakthrough infection in individuals with hybrid immunity. Aspects of figure include illustrations from NIH BioArt Source (bioart.niaid.nih.gov/bioart/463, bioart.niaid.nih.gov/bioart/481, bioart.niaid.nih.gov/bioart/313, bioart.niaid.nih.gov/bioart/98, bioart.niaid.nih.gov/bioart/379).

Notably, the protection in the hybrid immunity group was not solely attributable to humoral immunity. Hybrid-immunized individuals who remained uninfected during the follow-up period exhibited elevated IL-8 levels, which positively correlated with S100A8/A9, a neutrophil activation marker. These findings suggest an association between the IL-8–neutrophil axis and protection against breakthrough infection. Mechanistically, SARS-CoV-2 nucleocapsid proteins induced IL-8 production in monocyte-derived macrophages, enhancing neutrophil chemotaxis *in vitro* (**Figure 6**). This may reflect a state of sustained innate immune activation following prior infection. However, although SARS-CoV-2 structural proteins induced cytokine production in differentiated macrophages *in vitro*, similar innate immune activation may also occur following vaccination, particularly with mRNA-based vaccine formulations. Therefore, the observed cytokine responses may reflect combined effects of both prior infection and vaccination-induced innate immune stimulation. Importantly, the distribution of vaccine doses was comparable between individuals with and without breakthrough infection (**Supplementary Table 1**). In addition, vaccine dose was not significantly associated with IL-8 levels or the neutrophil activation markers S100A8/A9, whereas only a weak inverse correlation was observed with N-IgG titers (**Supplementary Table 2**). These findings suggest that the observed innate immune signatures are not primarily driven by differences in vaccination dose. S100A8/A9 is not unique to SARS-CoV-2 infection and has also been associated with innate immune activation in other viral infections and inflammatory conditions, including influenza (30, 31). Therefore, the elevated S100A8/A9 levels observed in this study may reflect a broader state of innate immune activation rather than a SARS-CoV-2-specific response.

This observation may be consistent with features of trained immunity, in which innate immune cells exhibit a form of memory following prior stimulation (32, 33). However, as our study did not directly assess the functional or epigenetic hallmarks of trained immunity, these findings should be interpreted with caution. Instead, our data primarily suggest sustained innate immune activation in individuals with hybrid immunity. Although such inflammatory response may be detrimental in certain contexts, they may also contribute to protection against breakthrough infections during periods of high viral circulation.

Sustained inflammation in cases of immunosuppression and advanced age has been associated with long COVID and delayed viral clearance (34). Evidence suggests that macrophages’ sustained activation, increased production of inflammatory cytokines, and their dissemination to the central nervous system, as well as prolonged activation and exhaustion of T cells, are key contributors (35). Notably, reports also suggest that the virus may persist long-term in the gastrointestinal tract, brain, and lymph nodes of recovered individuals (36). In such cases, virus-derived RNA and proteins remain in the body, leading to continued antigen presentation by macrophages and dendritic cells, causing sustained macrophage reactivation (37). In individuals with prior natural infection, subtle inflammation may persist even after recovery, remaining clinically silent but capable of triggering immune responses. Notably, 17 participants (65%) in the hybrid immunity group had been infected 6–12 months before sampling (**Supplementary Figure 5A**), suggesting a lingering inflammatory state and the possibility of an asymptomatic latent infection even in apparently healthy individuals. Given the timing of these infections, a substantial proportion likely occurred during the Omicron-dominant period, which may have influenced the observed immune profiles. Moreover, inflammatory proteins, such as IL-8, have short half-lives, rendering it unlikely that proteins produced during natural infection would remain detectable in the blood six months to one year later. Therefore, their persistence likely reflects either persistent infection or long-lived macrophages. Tissue-resident macrophages in the alveoli are known to survive for several months to years (38). Furthermore, due to symptom overlap between long COVID and autoimmune diseases, a potential association has been proposed (39), with evidence pointing to the possibility of prolonged persistence of inflammatory states. Autoantibodies are one of the causes of autoimmune diseases; they form immune complexes that lead to excessive activation of macrophages, release of IL-8, and neutrophil recruitment to the local site, thereby establishing an inflammatory amplification loop (40). In our study, IL-8 production was significantly increased in the hybrid immunity group that did not experience breakthrough infection (**Figure 2A**); however, no significant difference was observed between the hybrid immunity group as a whole and the high-vaccine-induced immunity group or the low-vaccine-induced immunity group (**Supplementary Figure 6A**). In contrast, the neutrophil activation marker S100A8/A9 levels were significantly increased across the entire hybrid immunity group (**Figure 5G**). Notably, among all measured cytokines and chemokines, only IL-8 production showed a significant positive correlation (**Supplementary Figure 6B**). This suggests that IL-8 may act as a central factor amplifying the autoantibody-induced inflammatory cycle.

Among the 26 individuals with a confirmed history of infection, excluding the two most recent cases, 24 cases had been infected by the following strains: the Alpha strain (1 case), Delta strain (1 case), Omicron BA.1/2 strain (5 cases), and Omicron BA.5 strain (17 cases) during the spread phase in Japan (**Supplementary Figure 5B**). Participants who received hybrid immunization through a combination of natural infection and vaccination during this period retained at least some neutralizing activity against the next-generation Omicron strains (XBB.1.16 and EG.5.1.1) (**Supplementary Figure 2A**), suggesting cross-reactive immunity activation.

Our study has some limitations. First, the definition of prior infection relied on anti-N IgG titers and self-reported diagnosis, which may underestimate subclinical infections. Second, the analysis was limited to serum-based immune markers without direct evaluation of cellular immune responses, including antigen-specific T cell function, within the study cohort. Therefore, the contribution of T cell-mediated immunity to protection against breakthrough infection could not be fully assessed. Third, the relatively small sample size and short follow-up period restrict conclusions regarding long-term protection. Fourth, a substantial proportion of participants with prior infection were likely infected during the Omicron-dominant period. As immune responses may vary depending on the infecting variant, the enhanced immunity observed in individuals with prior infection may partly reflect variant-specific immune imprinting rather than infection history alone. Due to the limited sample size, we were unable to perform a sufficiently powered stratified analysis based on the infecting variant. Fifth, although monocyte-derived macrophage differentiation was validated by flow cytometric analysis of macrophage-associated markers (**Supplementary Figure 4**), these *in vitro* differentiated cells may not fully recapitulate the phenotype and function of tissue-resident macrophages *in vivo*. Despite these limitations, our findings underscore the multifaceted nature of hybrid immunity, encompassing enhanced humoral breadth and sustained innate immune activation. With the ongoing evolution of the SARS-CoV-2 pandemic, understanding the synergy between adaptive and innate responses will be pivotal for guiding vaccine strategies and evaluating correlates of protection against future variants.

In conclusion, hybrid immunity offers superior protection against Omicron variants through sustained neutralizing antibody responses and persistent innate immune activation. Elevated IL-8 levels and neutrophil activation markers in hybrid-immunized individuals who avoided breakthrough infections suggest that innate immunity contributes to protection against specific variants. Our study provides a basis for further investigation into how hybrid immunity shapes long-term immune landscapes and may inform future vaccine design and public health strategies.

## Data Availability

The raw data supporting the conclusions of this article will be made available by the authors, without undue reservation.
