# Supplementary material for: Hybrid immunity from bivalent vaccination and prior infection enhances humoral and innate protection against Omicron XBB.1.16 and EG.5.1.1 variants in Japan
Source: Front Immunol. 2026 Jul 2;17:1807238. doi: 10.3389/fimmu.2026.1807238 (PMC13374799; doi:10.3389/fimmu.2026.1807238)
Supplement: Supplementary file 1 [file DataSheet1.pdf]

Supplementary Material

1 Supplementary Figures

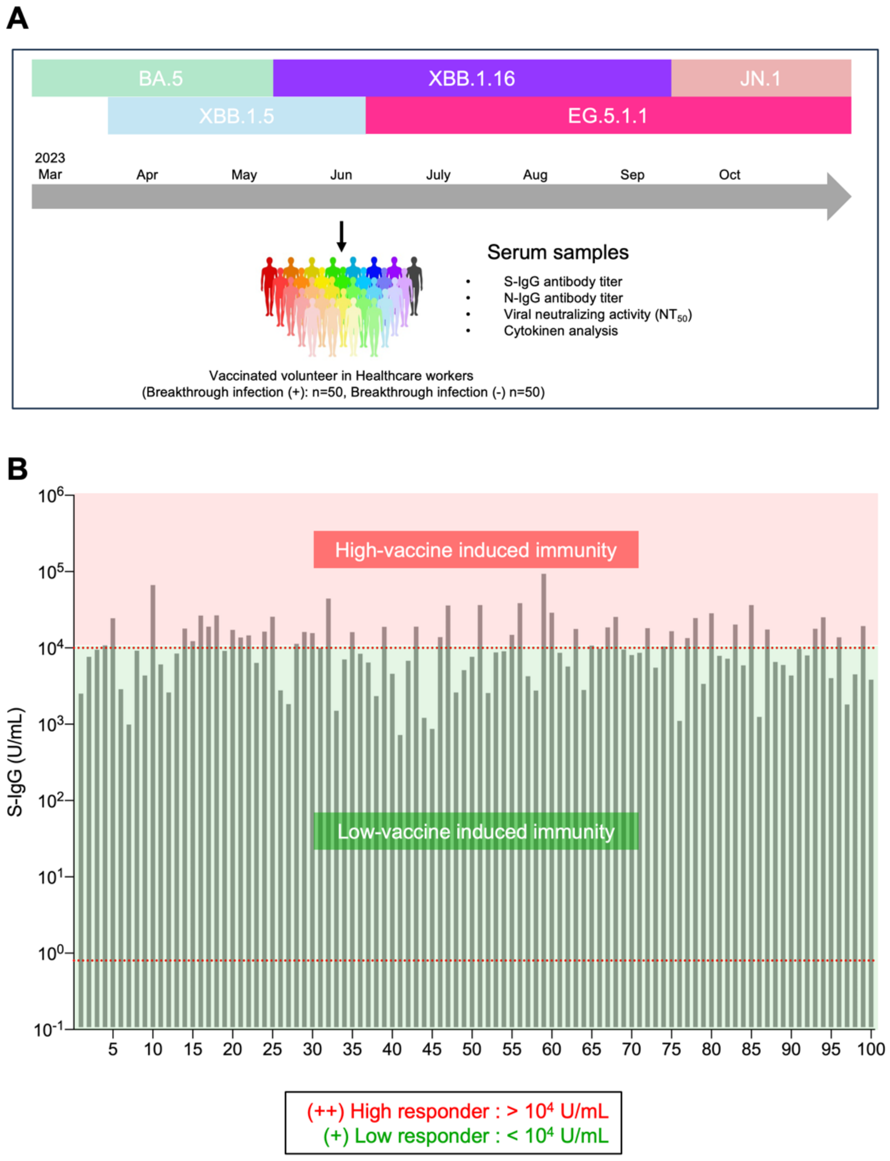

Supplementary Figure 1. Schematic study design.

(A) Study design schematic and comparison of the neutralizing and anti-nucleocapsid antibody titers among 100 participants including 50 breakthrough infection cases and 50 no-breakthrough infection cases. **(B)** Comparison of the anti-spike antibody titers among 100 participants. Participants were classified into high and low-vaccine-induced immunity groups based on an S-IgG antibody titer of 10^4 U/mL.

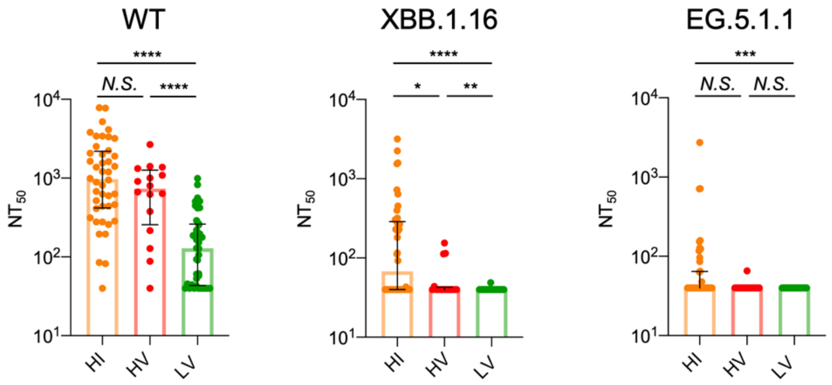

Supplementary Figure 2. Hybrid immunization induced by bivalent vaccination and natural infection maintains effective neutralizing antibody activity against the Omicron variant.

Comparison of neutralizing antibody titers among three groups against WT, Omicron XBB.1.16, and Omicron EG.5.1.1 variants. Bar graphs indicate the median titers, and the I-shaped bars denote interquartile ranges. Statistical significance was determined using the Mann–Whitney U test (N.S.: not significant; * $P < 0.05$; ** $P < 0.01$; *** $P < 0.001$; **** $P < 0.0001$). NT_{50}, 50% neutralization titer; HI, Hybrid Immunity; HV, High-vaccine induced immunity; LV, Low-vaccine induced immunity.

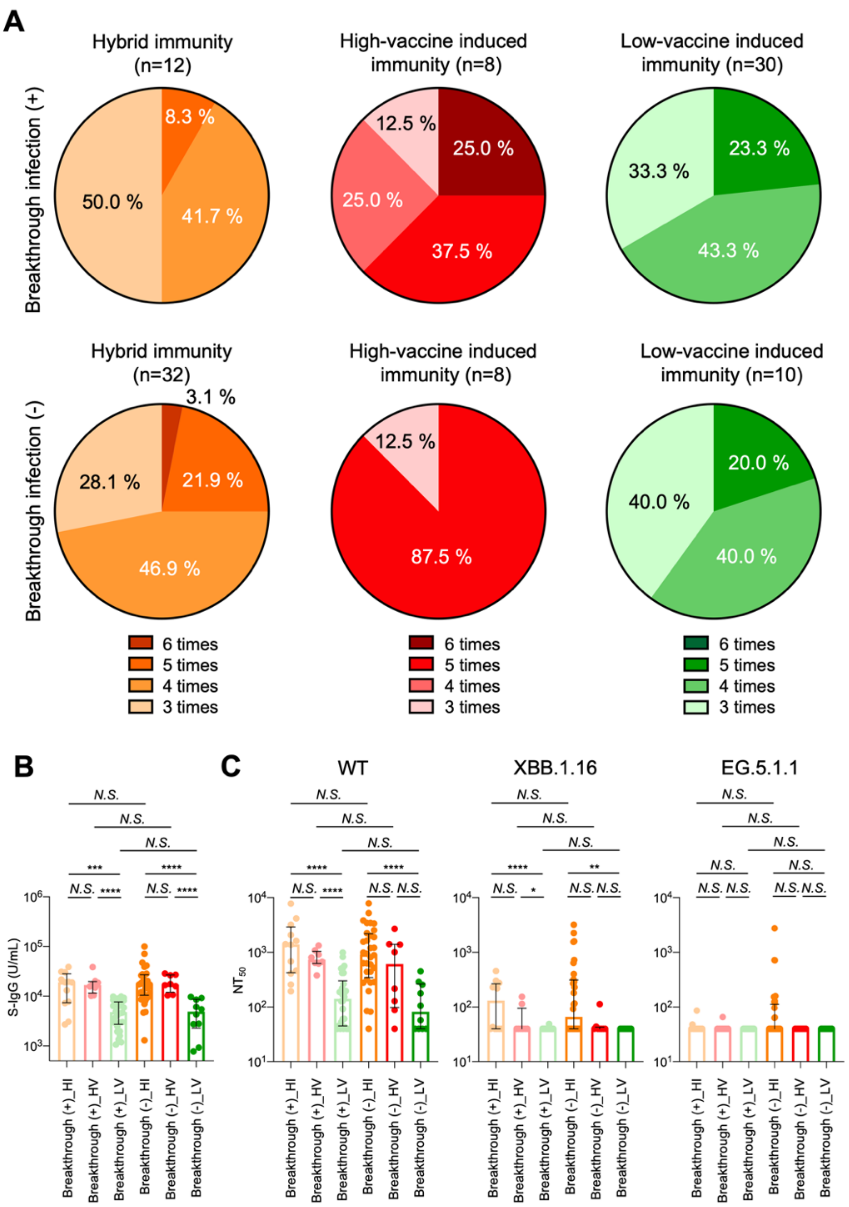

Supplementary Figure 3. Adaptive immunity does not contribute to the prevention of breakthrough infection.

(A) Vaccination status across the three groups was compared based on the presence or absence of breakthrough infections. The number of vaccinations and the number of breakthrough infections were calculated using a self-reported questionnaire survey. Comparison of the anti S-IgG titers **(B)** and neutralizing antibody titers among the six groups **(C)**. Neutralizing antibody titers against WT, Omicron XBB.1.16, and Omicron EG.5.1.1 variants. Bar graphs indicate the median titers, and the I-shaped bars denote interquartile ranges. Statistical significance was determined using the Mann–Whitney U test. (N.S.: not significant; * P < 0·05; ** P < 0.01; *** P < 0·001; **** P < 0·0001). HI, Hybrid Immunity; HR, High Responder; LR, Low Responder

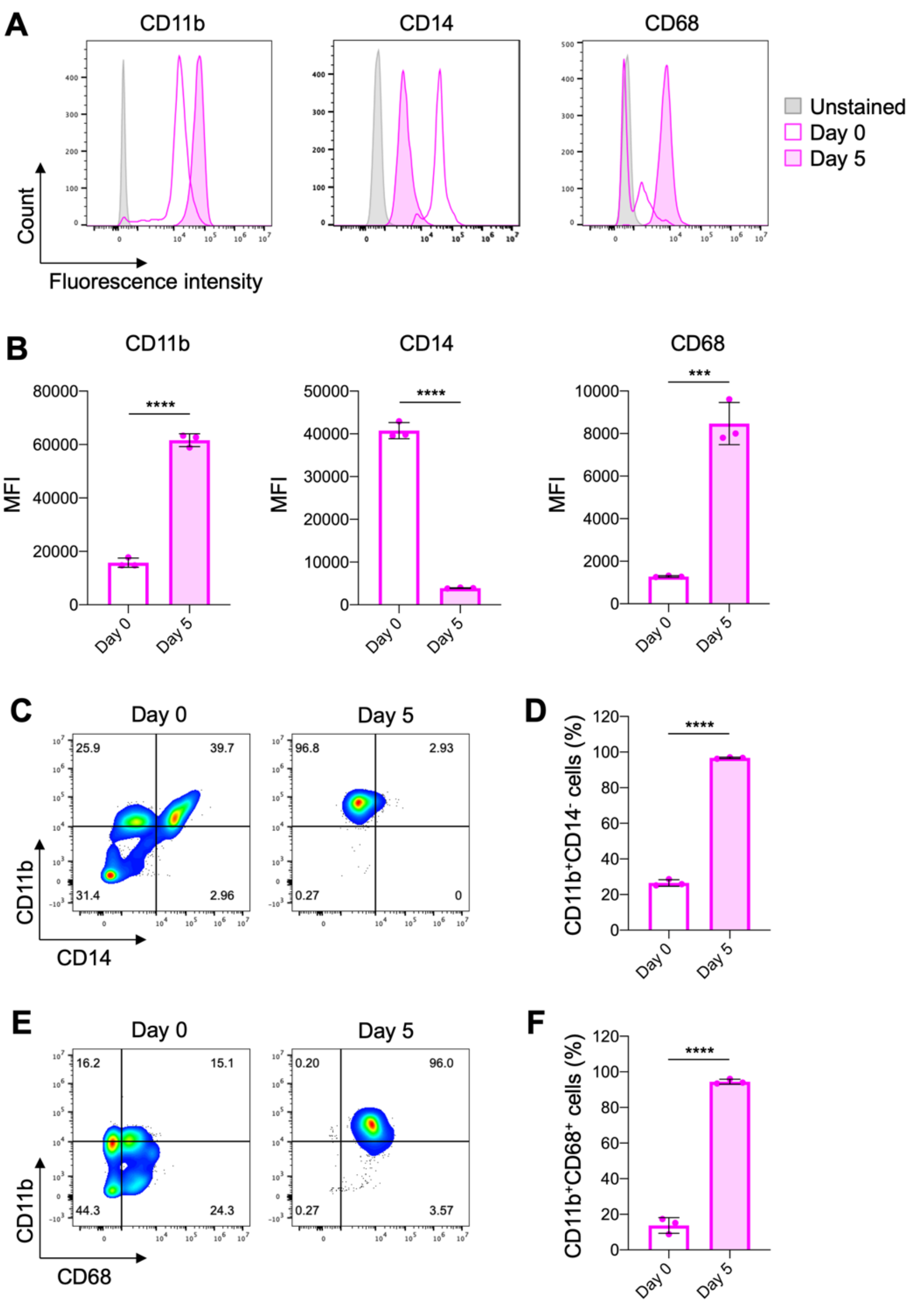

Supplementary Figure 4. Differentiation of MDMs.

Human monocytes were differentiated following 5 days of stimulation with 100 ng/mL GM-CSF. **(A)** Representative histogram of CD11b, CD14 and CD68 expression in unstained, unstimulated (day 0) and stimulated (day 5). **(B)** Comparison of the mean fluorescence intensity (MFI) of CD11b, CD14 and CD68 between day 0 and day 5. (**C**) (**D**) Comparison of $CD11b^{+}CD14^{-}$ cells between day 0 and day 5. (**E**) (**F**) Comparison of $CD11b^{+}CD68^{+}$ cells between day 0 and day 5. Bar graphs indicate the mean, and the I-shaped bars denote standard deviations. Statistical significance was determined using the unpaired t-test (*** P < 0·001; **** P < 0·0001).

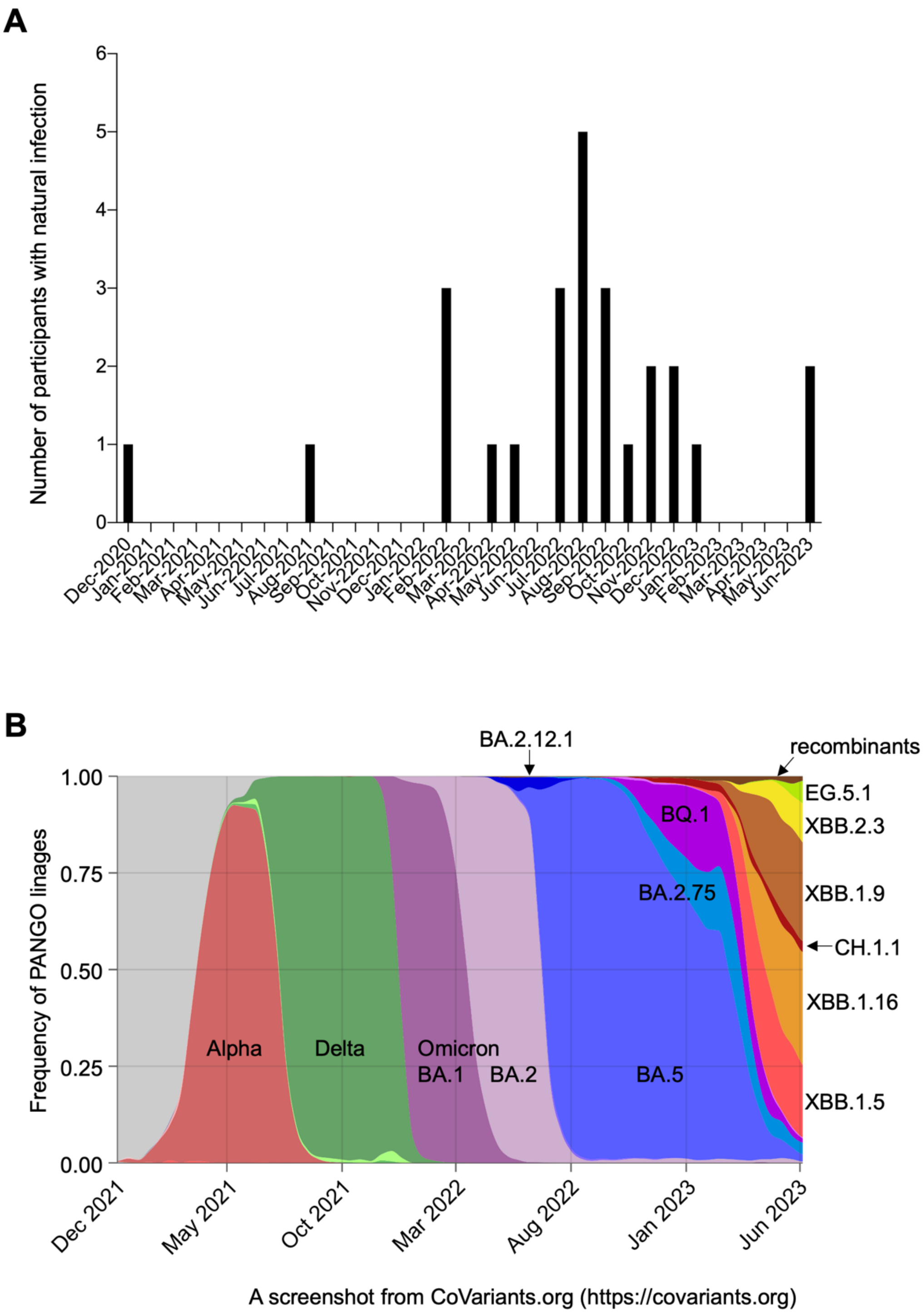

Supplementary Figure 5. Timing of infection in naturally infected individuals and the dynamic evolution of SARS-CoV-2 variants in Japan.

(A) The number of participants with natural infections from Dec 2021 to Jun 2023. **(B)** Trends in the frequency of VOC and PANGO lineages in Japan from Dec 2021 to Jun 2023. A screenshot from CoVariants.org (https://covariants.org) data was used. VOC, variants of concern.

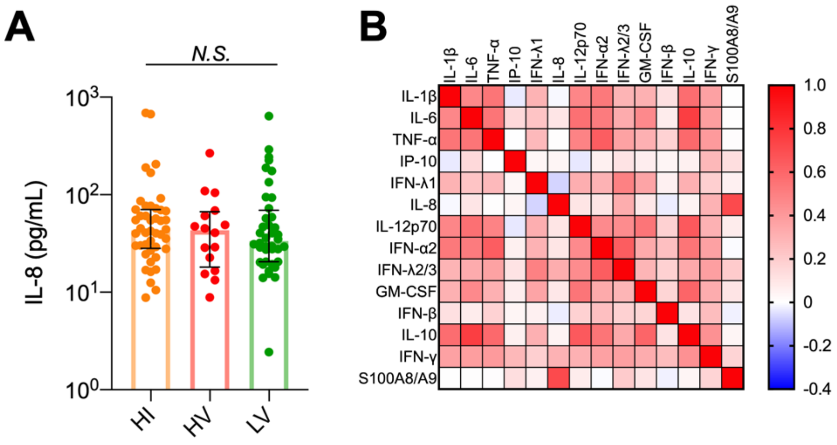

Supplemental Figure 6. Correlation analysis.

(A) Comparison of IL-8 serum levels among the three groups. **(B)** A heatmap of the Pearson's correlation matrix of the relationship between serum S100A8/A9 and serum cytokine levels. Red demonstrates positive correlation, and blue represents negative correlation. Bar graphs indicate the median titers, and the I-shaped bars denote interquartile ranges. Statistical significance was determined using the Mann–Whitney U test (N.S.: not significant).

2 Supplementary Tables

Supplementary Table S1. Distribution of vaccine doses according to breakthrough infection status.

Vaccine doses	Breakthrough (+) [a]	Breakthrough (-) [a]	P value [b]
3 doses	34 %	28 %	
4 doses	40 %	38 %	
5 doses	22 %	32 %	
6 doses	4 %	2 %	0.6650

[a] Values are presented as number (%). [b] Differences in the distribution of vaccine doses between individuals with and without breakthrough infection were assessed using the Chi-square test. No significant differences were observed between groups.

Supplementary Table S2. Association between vaccine dose and immune logical parameters.

Variable	Correlation coefficient (r) [a]	P value [b]
IL-8	-0.02108	0.8351
S100A8/A9	-0.09343	0.3552
N-IgG titer	-0.2057	0.0400

[a] Values represent Pearson correlation coefficients. [b] P-value (two-tailed). N = 100.
